# Unilateral Central Retinal Vein Occlusion in a Young Adult With Latent Syphilis

**DOI:** 10.7759/cureus.99103

**Published:** 2025-12-13

**Authors:** Li Lian Chew, Tajunisah Iqbal, Nazirah Ibrahim, Kiew Ing Tiong

**Affiliations:** 1 Department of Ophthalmology, Faculty of Medicine, UM Eye Research Centre, University Malaya, Kuala Lumpur, MYS; 2 Department of Ophthalmology, Hospital Ampang, Selangor, MYS; 3 Department of Ophthalmology, Sarawak General Hospital, Sarawak, MYS

**Keywords:** central retinal vein occlusion, partial recovery, retinal vein occlusion, syphilis, unilateral, young

## Abstract

Central retinal vein occlusion (CRVO) is a significant retinal vascular disease that commonly affects the elderly. Given its rarity among the younger population, its presence should alert clinicians to the possibility of a serious systemic disease. We highlight a rare case of CRVO in a young adult with latent syphilis. Our patient is a 24-year-old male who presented with acute, right eye painless blurring of vision for one day. His right and left visual acuity (VA) were hand movement and 6/6, respectively. Relative afferent pupillary defect (RAPD) was negative. Right fundus examination revealed a hyperemic optic disc with dilated tortuous retinal vessels and multiple flame-shaped, dot, and blot hemorrhages. Laboratory investigations showed a positive *Treponema pallidum *particle agglutination (TPPA) and reactive rapid plasma reagin (RPR) with a titer of 1:16 and a weakly positive anti-cardiolipin antibody, which became negative after repeating at 16 weeks and 32 weeks. Cerebrospinal fluid (CSF) Venereal Disease Research Laboratory (VDRL) test was non-reactive. The patient was diagnosed with central retinal vein occlusion (CRVO) secondary to latent syphilis. A total of three doses of intramuscular benzathine penicillin 2.4 MU were administered weekly. Post-treatment, the patient's right eye VA improved dramatically to 6/36 over a 4-week period. As syphilis is a curable disease, prompt recognition and management are crucial in achieving a good vision outcome and avoiding lifelong devastating blindness, as reflected in this case.

## Introduction

Central retinal vein occlusion (CRVO) is a disease that predominantly affects the older population and is relatively rare among the young [1]. CRVO in the younger population may serve as an early marker of a serious systemic disease, for which rarer causes such as infection, inflammation, or blood dyscrasia should be excluded [2]. Here, we report an unusual case of CRVO in a young male with latent syphilis. Syphilis is known as the great imitator, mimicking numerous diseases, as it can affect any structure of the eye at any stage of the disease. It is important not to miss ocular syphilis, as it is a curable condition, and early diagnosis and treatment may prevent lifelong irreversible ocular morbidity.

## Case presentation

A 24-year-old Malay male, a non-smoker with no known medical illness, presented with complaints of a sudden onset of unilateral, painless, generalized blurring of vision for one day. The blurring of vision was not associated with floaters, flashes of light, or headache. Further history revealed past homosexual behaviour.

**Figure 1:**
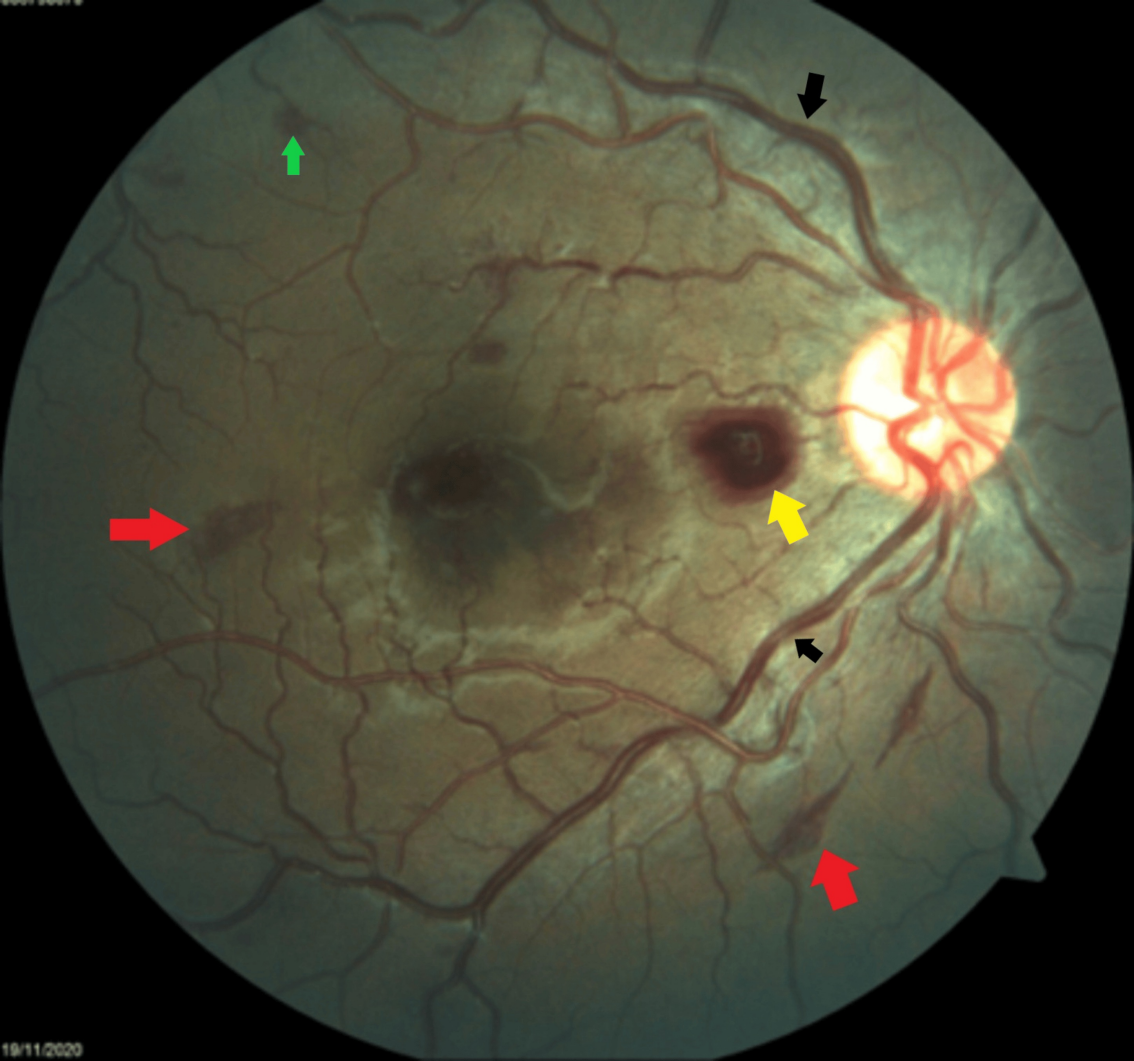
Right eye fundus showing tortuous and dilated blood vessels (black arrows) in all four quadrants with flame-shaped (red-arrows), dot (green arrow), and blot haemorrhages (yellow arrow).

**Figure 2:**
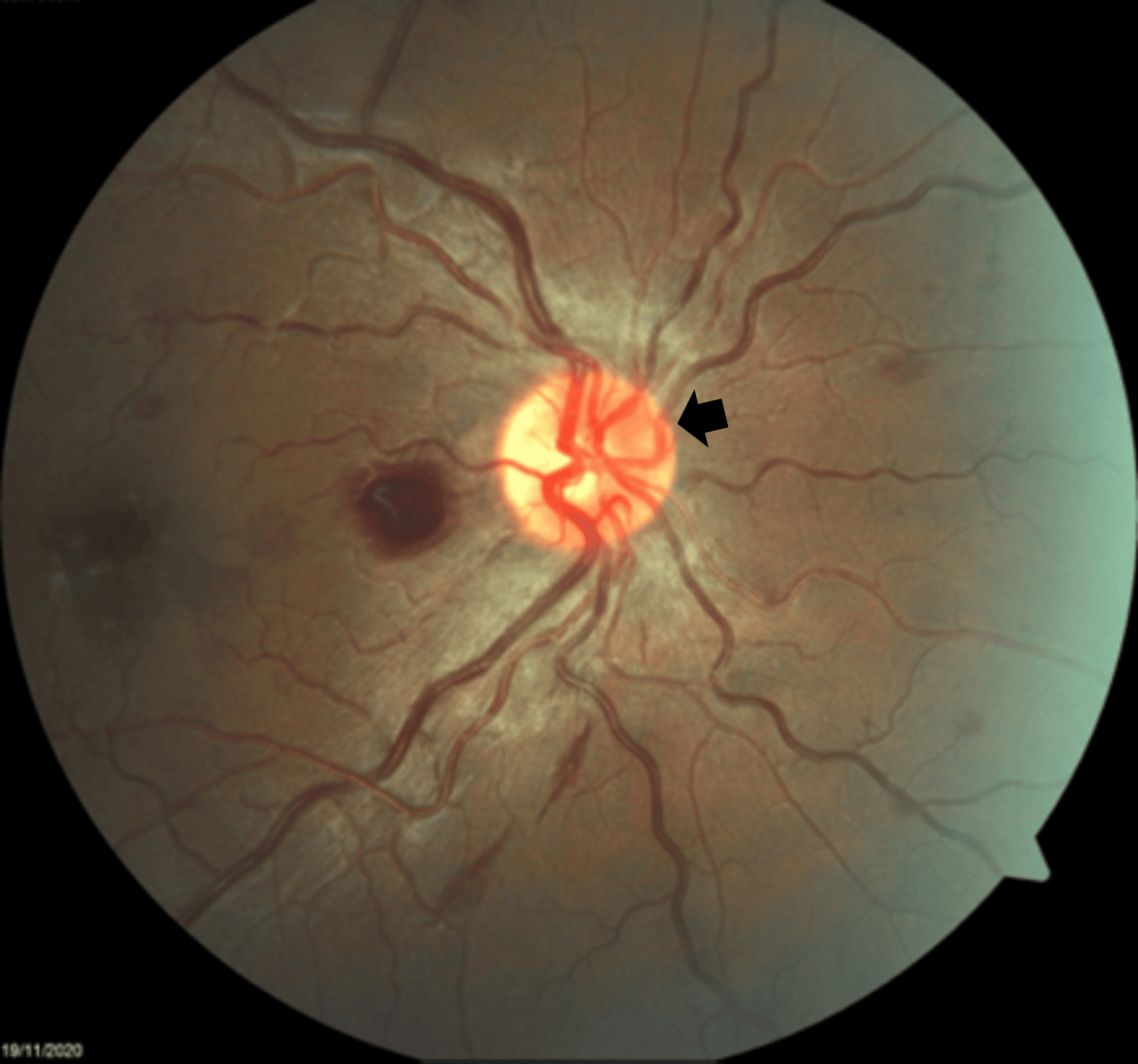
Right eye fundus showing hyperaemic optic disc (black arrow).

On presentation, his best-corrected visual acuity (BCVA) was hand movement in the right eye and 6/6 in the left eye. A relative afferent pupillary defect (RAPD) was not present. Anterior segment examination of both eyes was normal. Intraocular pressure was 10 mmHg bilaterally. Fundus examination of the right eye revealed a hyperemic optic disc with a cup-disc ratio of 0.3, with dilated and tortuous blood vessels in all four quadrants, with multiple flame-shaped haemorrhages, and dot and blot haemorrhages, as shown in Figure 1 and Figure 2. Otherwise, there was no vitritis, no sheathing of vessels, and no retinitis or cotton wool spots. The fundus of the left eye was unremarkable with a pink optic disc with a cup-disc ratio of 0.3 and normal retinal vessels. Optical coherence tomography of the right eye showed intraretinal fluid with cystic macular oedema involving the fovea. Fluorescein fundus angiography was not carried out due to a clinical setting limitation. Otherwise, the patient’s general systemic examination and vital signs were normal. His blood pressure was 128/70 mmHg, capillary blood glucose was 5.0 mmol/L, heart rate was 70 bpm, and electrocardiography showed sinus rhythm.

Blood investigations, including complete blood count, erythrocyte sedimentation rate (ESR), renal function, fasting blood sugar, fasting lipid profile, coagulation profile, anti-nuclear antibodies (ANA), rheumatoid factor, protein C, protein S, anti-beta-2 glycoprotein immunoglobulin, human immunodeficiency virus (HIV), hepatitis B, hepatitis C virus serology, and plasma homocystein level were normal. Contrasted computerized tomography of the brain, orbit, and angiogram were unremarkable. Ultrasound carotid doppler and echocardiogram showed normal findings. Anti-cardiolipin antibody was weakly positive at 36.6 U/ml but was negative on repeated samples at 16 weeks and 32 weeks by the rheumatology team. Rapid plasma reagin (RPR) was reactive with a titre of 1:16, and *Treponema pallidum *particle agglutination (TPPA) was positive. With the co-management of the neuromedical team, a lumbar puncture was performed. He was initially given intravenous benzylpenicillin.

**Figure 3:**
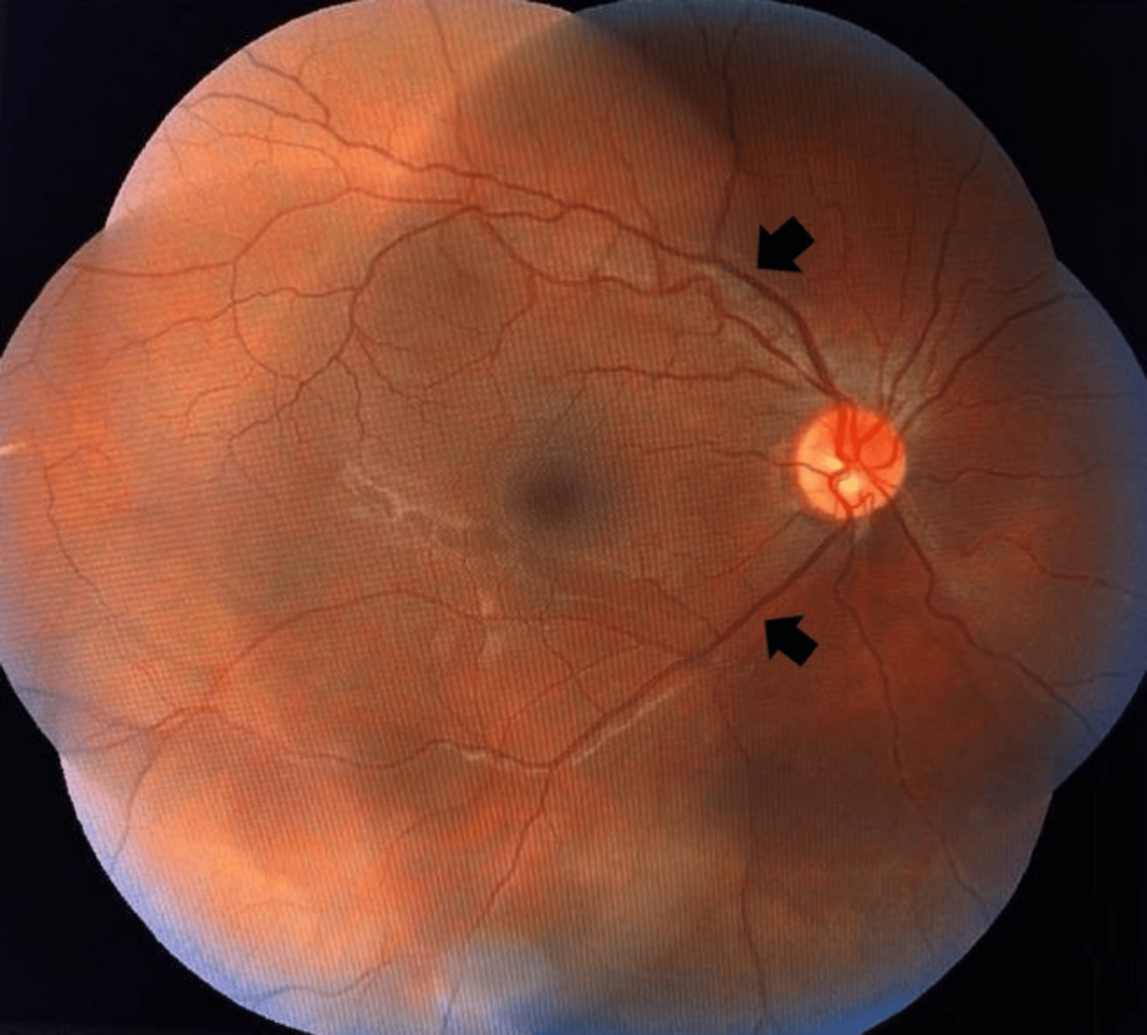
Right eye fundus showing resolved dot blot hemorrhages with reduced tortuosity of vessels (black arrows) 2 months after the completion of the treatment.

**Figure 4:**
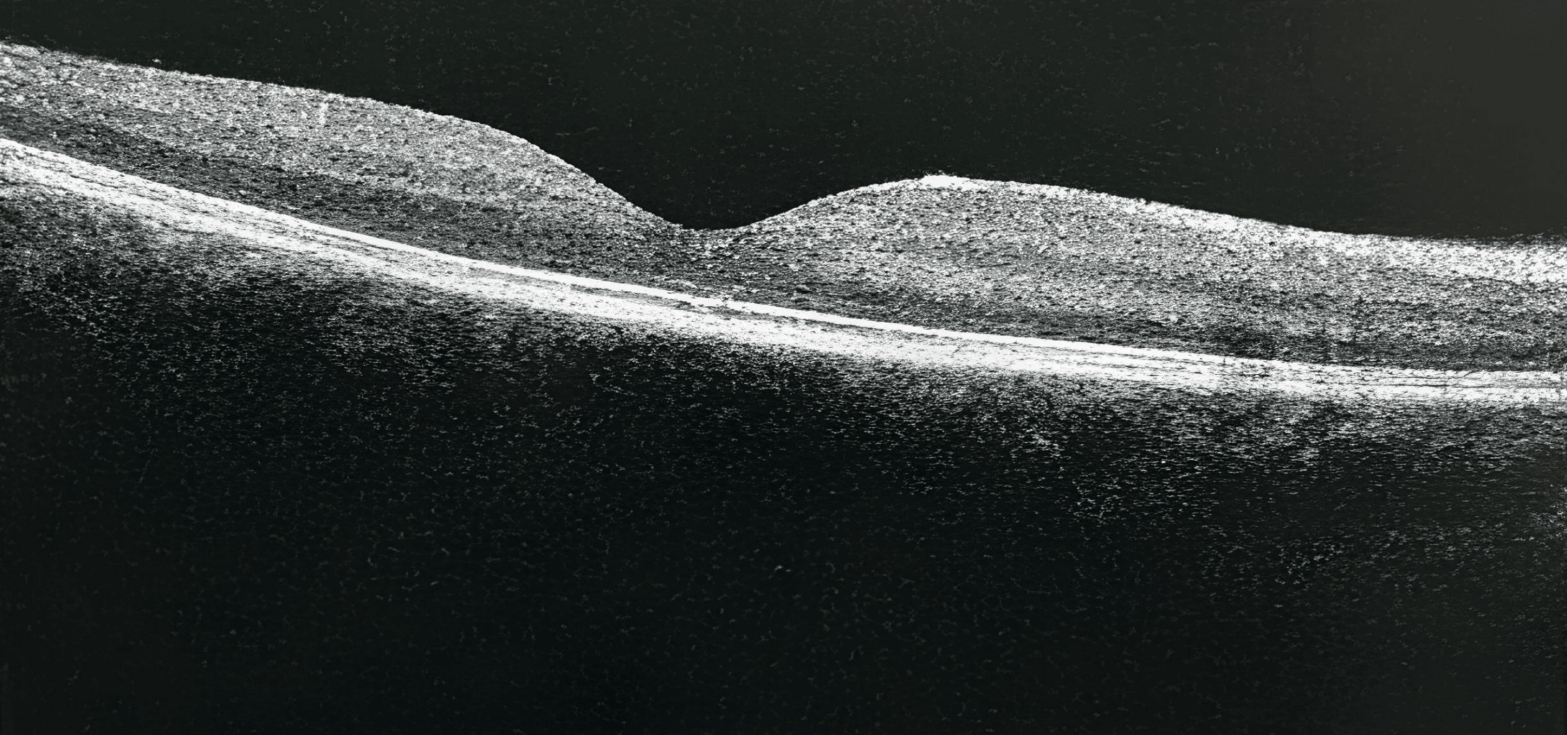
Optical coherence tomography of the right eye at 6 months of follow-up showing resolved cystic macular oedema

However, as the cerebrospinal fluid (CSF) Venereal Disease Research Laboratory (VDRL) test came back non-reactive, and other CSF results were normal, including normal opening pressure (12 cm H₂O), clear CSF appearance, normal cell count (1 WBCs/µL), normal protein (20mg/dL), normal glucose (3.5 mmol/L), negative Gram stain, negative culture, and negative India ink, we treated him for latent syphilis. Subsequently, a total of three doses of intramuscular benzathine penicillin G 2.4 million units injections were administered weekly for three consecutive weeks. Upon completion of treatment after 1 month, the patient’s BCVA in his right eye improved dramatically from hand movement to 6/36. Fundus examination also showed regression of the scattered dot blot haemorrhages 2 months after completion of treatment, as demonstrated in Figure 3. During his latest follow-up (6 months post-discharge), he did not show worsening of symptoms, and optical coherence tomography showed resolved cystic macular oedema of the right eye, as can be seen in Figure 4.

## Discussion

Central retinal vein occlusion (CRVO) is mainly diagnosed clinically, supported by hallmark findings on a dilated fundus examination. Patients typically present with sudden, painless vision loss or blurring in one eye. Fundoscopy shows the classic ‘blood and thunder’ appearance, including widespread intraretinal hemorrhages, dilated and tortuous veins, cotton wool spots, and optic disc edema. CRVO may affect an individual at any age, but it is predominantly diagnosed among the elderly, specifically those over 50 years of age, and is relatively rare among younger individuals [1]. Out of all the CRVO cases, only 10-15% are reported in patients under 40 years of age [3,4]. CRVO among elderly patients is often associated with underlying conditions such as diabetes mellitus, hypertension, or atherosclerotic vascular disease [3]. Although such classical risk factors were also reported among young patients with CRVO, the incidence rates are much less prevalent when compared with their older counterparts [4]. Among young patients with CRVO under 50 years of age, only 23% had hypertension (compared to 62% in older adults), 3-9% had diabetes mellitus, and 0-9% had hyperlipidemia. Hence, the exact cause among the majority of the young CRVO patients remains unclear.

The non-classical risk factors associated with CRVO in the younger population include hypercoagulable states (polycythemia, protein C or protein S deficiency, antiphospholipid syndrome, collagen vascular disease, myeloproliferative disorder), syphilis, sarcoidosis, Behcet’s disease, and HIV infection [2,3,5]. A study conducted by Rothman et al showed that of 36 younger patients (below the age of 50) with CRVO, 21 patients (58%) had at least one identifiable non-classical risk factor for CRVO. These include the use of hormonal oral contraceptives or hormone-secreting intrauterine device, postpartum period, and hypercoagulopathy conditions (methylenetetrahydrofolate reductase mutation, factor V Leiden, intense exercises, sarcoidosis, systemic lupus erythematosus, autoimmune hepatitis, Bechet’s disease, and use of tyrosine kinase inhibitor (sorafenib)). As such, in a young patient with CRVO, a comprehensive systemic workup is crucial in order to not miss any significant systemic conditions that predispose them to CRVO.

It was observed that both younger and older patients with CRVO share identical fundus findings such as dilated tortuous retinal veins, disc swelling, and peripheral retinal haemorrhages [5]. Despite that, young patients with CRVO tend to have milder, nonischemic conditions that are associated with better visual outcomes in contrast to the older population [3]. The exact pathogenesis of CRVO remains obscure; however, intraluminal thrombus formation was believed to be the definite underlying pathogenesis in all CRVO cases. Thrombosis has been postulated to occur from a combination of four primary mechanisms: degenerative/inflammatory vessel wall changes, hypercoagulable state, abnormal blood flow, and apposition of sclerotic vessels in a common adventitial sheath [3].

In ocular syphilis, retinal veins, arteries, arterioles, and capillaries can all be affected [6]. It can range from increased vascular tortuosity, perivascular exudation and fibrosis, to obliteration of vessels from an occlusive vasculitis, or can even masquerade as a branch retinal vein occlusion [6,7]. Retinal vasculitis causes vascular occlusion through a thrombotic or obliterative mechanism and may occur as a result of infections, systemic immune-mediated disease, or idiopathic causes [8]. Among the infections associated with retinal vasculitis are tuberculosis, syphilis, toxoplasma, herpes viruses, and Lyme’s disease. In ocular syphilis, infective retinal vasculitis may occur as a result of direct invasion of *Treponema pallidum* or due to deposition of immune complexes on the endothelium of blood vessels [9].

Syphilis is a highly infectious, multisystem bacterial infection caused by *Treponema pallidum*. It is divided into four stages: primary syphilis, secondary syphilis, latent syphilis, and tertiary syphilis [10]. It can affect any structure of the eye at any stage. Primary syphilis is most often associated with a chancre at the site of inoculation, such as the eyelid or conjunctiva [10]. Secondary syphilis can manifest as rashes involving the eyelid, conjunctivitis, episcleritis, scleritis, keratitis, iridocyclitis, and anterior uveitis. In late secondary syphilis, there can be posterior segment involvement such as vitritis, necrotising retinitis, retinal vasculitis, optic neuritis, and chorioretinitis. Latent syphilis is a stage that is detected only through serological testing without any clinical manifestations. If untreated, it may progress to tertiary syphilis, giving rise to serious neurological and cardiovascular complications. Ocular manifestations of tertiary syphilis include gumma of the eyelid, madrosis, keratitis, arterial occlusive disease, retinal detachment, and optic neuritis.

In most cases, syphilis is curable with a single dose of intramuscular benzathine penicillin G 2.4 million units [6]. Our patient had latent syphilis for which the recommended treatment is three doses of intramuscular Benzathine Penicillin G 2.4 million units given at weekly intervals. Alternatively, in patients with penicillin allergy, they may be offered a course of oral doxycycline (100 mg twice daily) up to 4 weeks [6]. Syphilis is a curable disease; therefore, early recognition and initiation of treatment with appropriate antibiotics can render a cure and avoid devastating ocular morbidity, including blindness and other life-threatening systemic complications.

## Conclusions

We report a rare case of central retinal vein occlusion (CRVO) in a young adult with latent syphilis. As syphilis is a curable disease, prompt recognition and management are important to achieve a good vision outcome and prevent devastating blindness, as demonstrated in our case.

## References

[1] Primo S (1990). Central retinal vein occlusion in a young patient with seropositive syphilis. J Am Optom Assoc.

[2] Rothman AL, Thomas AS, Khan K, Fekrat S (2019). Central retinal vein occlusion in young individuals: a comparison of risk factors and clinical outcomes. Retina.

[3] Bhagat N, Goldberg MF, Gascon P, Bell W, Haberman J, Zarbin MA (1999). Central retinal vein occlusion: review of management. Eur J Ophthalmol.

[4] McGrath MA, Wechsler F, Hunyor AB, Penny R (1978). Systemic factors contributory to retinal vein occlusion. Arch Intern Med.

[5] Sinawat S, Bunyavee C, Ratanapakorn T, Sinawat S, Laovirojjanakul W, Yospaiboon Y (2017). Systemic abnormalities associated with retinal vein occlusion in young patients. Clin Ophthalmol.

[6] Kiss S, Damico FM, Young LH (2005). Ocular manifestations and treatment of syphilis. Semin Ophthalmol.

[7] Lobes LA Jr, Folk JC (1981). Syphilitic phlebitis simulating branch vein occlusion. Ann Ophthalmol.

[8] Ku JH, Ali A, Suhler EB, Choi D, Rosenbaum JT (2012). Characteristics and visual outcome of patients with retinal vasculitis. Arch Ophthalmol.

[9] (2024). Central retinal vein occlusion due to retinal vasculitis. https://www.uveitis.org/wp-content/uploads/2017/05/vasculitis.pdf.

[10] Koundanya VV, Tripathy K (2025 Jan-). Syphilis ocular manifestations. StatPearls [Internet].

